# Efficacy of a Decision‐Making Aid About Homeopathy in Patients With Cancer: A Single‐Arm, Pre–Post Observational Study

**DOI:** 10.1002/hsr2.72943

**Published:** 2026-07-30

**Authors:** Maximilian Gimbel, C. Stoll, St. Fuxius, F. J. Prott, K. Münstedt, B. Zomorodbakhsch, Sandra Wittmann, Karin Kastrati, J. Hübner

**Affiliations:** ^1^ Klinik für Innere Medizin II, Universitätsklinikum Jena, Am Klinikum 1 Jena Germany; ^2^ Innere Medizin/Onkologie Klinik Herzoghöhe Bayreuth Germany; ^3^ Onkologische Schwerpunktpraxis Heidelberg Germany; ^4^ RNS Gemeinschaftspraxis Wiesbaden Germany; ^5^ Ortenau Klinikum Offenburg‐Kehl Offenburg Germany; ^6^ üBAG/MVZ Onkologische Kooperation Harz Offenburg Germany; ^7^ Klinikfür Onkologie, Hämatologie und Palliativmedizin HeliosDr. Horst Schmidt Wiesbaden Germany; ^8^ Nierenkrebs‐Netzwerk Wölfersheim Germany

**Keywords:** cancer, decision aid, homeopathy, integrative medicine

## Abstract

**Background and Aims:**

Despite lacking evidence, homeopathy continues to enjoy high levels of usage and general acceptance. Particularly concerning the treatment of cancer, this can pose risks, especially when it leads to delay or replacement of evidence‐based treatments. The goal was to assess informed decision‐making and analyze outcomes.

**Methods:**

The decision aid summarized key aspects of homeopathy, including its definition, distinction from naturopathy, production, approval, scientific evidence, placebo effects, use in cancer, side effects, and insurance coverage. Therefore, we conducted a single arm, pre‐post observational survey. A questionnaire assessed knowledge, attitudes, and use of homeopathy before and after reading the aid. It included demographic data, topic‐specific knowledge, use for certain medical conditions, and an evaluation section. A total of 123 adult cancer patients were recruited from oncology centers across Germany (2022), with no age restrictions. Changes in knowledge and attitudes were analyzed.

**Results:**

Participants significantly revised their views on homeopathy for most medical conditions; beliefs about its use for psychological stress remained unchanged. Knowledge about placebo effects and potentization improved significantly, whereas understanding of side effects did not. Prior use of homeopathy influenced post‐intervention decisions. Educational background and socioeconomic status showed no significant effects, demonstrating broad applicability of the intervention. Ordinal regression analyzes indicated that participants with lower prior knowledge benefited more, particularly regarding “potentization” and “placebo difference.” Post‐intervention knowledge gains varied by prior knowledge and gender, with less‐informed participants and male respondents benefiting more. Most participants felt well informed, gained new insights, and would recommend the material.

**Conclusion:**

The decision aid demonstrated potential to support evidence‐based decision‐making among oncology patients.

However, heterogeneity in outcomes indicates the influence of unmeasured factors, such as belief systems or emotional needs.

Future research should explore tailoring decision aids to gender‐ and knowledge‐levels. Integrating such tools into patient communication could enhance informed choice where alternative healing methods remain prevalent.

**Abbreviations, Definitions and Symbols**
CAR‐T cell therapy: Cancer treatment therapy
Placebo difference: The distinction between symptom improvement caused by expectations and context
Potentization: Homeopathic process of repeated dilution and shaking, believed within homeopathy to increase a remedy's effect
*p*‐value: Upper‐tail probability of the test statistic assuming that the null hypothesis is true
SPSS: Statistical package for social sciences (statistical data analysis software)

## 1 Introduction

Homeopathy is one of the most debated topics in the German healthcare system. Many patients use this form of therapy and are often convinced of its effectiveness [1], despite a lack of evidence [2]. This poses risks for oncology patients, as effective therapies may be delayed or interrupted due to homeopathy [3]. Therefore, it is important to examine homeopathic therapies in oncology more closely and to determine the reasons for such therapy decisions in patients.

A variety of factors influence patients' use of homeopathy, including cultural beliefs, emotional motivations, and personal experiences with conventional medicine. Some patients seek alternative treatments due to dissatisfaction with standard care, fear of side effects, or a general preference for natural remedies. Additionally, psychosocial influences, such as recommendations from family members, social circles, or online communities, contribute to the decision‐making process. Understanding these factors is crucial to designing more effective patient education strategies.

One important factor to consider is the cost of homeopathy. In 2019, the cost for statutory health insurance was approximately 20 million euros [4]. While it is not covered by the standard benefits package, about two‐thirds of the health insurance companies offer homeopathic treatments as additional services [5]. While this amount may not seem significant compared to the total drug expenditures of 40 billion euros, the money saved could be used to provide CAR‐T cell therapy for 62 patients (up to 320,000 euros per patient) [6].

However, budget allocation in healthcare is a complex issue, involving competing priorities across various sectors. While critics argue that homeopathy drains funds from evidence‐based treatments, others see value in patient satisfaction and psychological well‐being associated with complementary treatments. A more nuanced approach to resource distribution is necessary to balance these perspectives.

Another important aspect related to homeopathy is the continuing education requirements for doctors. The additional qualification in homeopathy allows doctors to improve the diagnosis and prescription of homeopathic remedies. In 2022, the German Medical Association responded to this issue and decided by a clear majority to remove the additional qualification from the continuing education requirements. Thirteen out of 17 state medical associations decided not to adopt the additional qualification in their state laws [7]. This decision reflects broader ethical concerns regarding the role of homeopathy in treating vulnerable patients, particularly those with serious illnesses such as cancer. While patient autonomy is a fundamental principle of medical ethics, physicians also have a duty to ensure that patients receive scientifically supported treatments. The integration of homeopathy into oncology raises questions about informed consent, medical responsibility, and potential conflicts of interest among healthcare providers.

The registration and approval of homeopathic drugs also raise questions. The Federal Institute for Drugs and Medical Devices has registered 3465 drugs and approved 1127 drugs [8], but efficacy studies are not required, as they are for other drugs [9].

This regulatory gap has led to diverging opinions among stakeholders, including policymakers, healthcare providers, and pharmaceutical companies. Some view homeopathies as a patient‐centered option that promotes holistic care, while others see it as a placebo‐based practice that should not receive institutional support. Understanding these differing attitudes is essential to shaping future policies on integrative medicine.

Despite these criticisms, many doctors and patients still hold onto homeopathy. A study conducted in 2018 showed that about 40% of the population used complementary and alternative medicine [10], including homeopathy, in the past year. Germany ranks first in Europe in this regard, with the European average being 25%. In Germany, 6500 doctors have the additional qualification in homeopathy [11].

The persistent demand for homeopathic treatments highlights the need for balanced, evidence‐based patient education rather than outright rejection or endorsement.

Patients want to be more involved in their therapy planning, and it is important to understand which factors influence decision‐making in illness situations and whether decision aids can help better educate patients about their situation and treatment options. Several studies have shown that decision aids can improve participation in therapy and the quality of decisions [12]. To our knowledge, no study has been published that examines the influence of a decision aid on the topic of homeopathy in cancer patients. Given the potential risks associated with delaying or interrupting evidence‐based cancer treatments, studying the role of homeopathy in oncology patients is particularly relevant. Cancer is a life‐threatening disease where treatment delays can significantly impact survival rates and patient outcomes. Decision aids can help patients critically evaluate alternative treatments and make informed choices that align with medical guidelines while respecting their personal preferences.

This study aims to capture the patient's perspective and focuses on the influence of patient information. With the help of a decision aid, this study examines which information on homeopathy is of particular interest and influences the decision‐making of patients. By providing structured, unbiased information, decision aids can serve as a crucial tool in improving patient awareness and ensuring that treatment choices are based on sound medical principles rather than misinformation or external pressure.

## 2 Methods

### 2.1 Decision Aid

The decision aid consisted of several parts:

1. Definition of homeopathy
2. Difference between naturopathy and homeopathy
3. Manufacturing process of homeopathic remedies
4. Approval and registration
5. Evidence from scientific investigations on homeopathy
6. Placebo and homeopathy
7. Homeopathy for cancer
8. Side effects of homeopathy
9. Cost coverage by health insurance companies

The decision aid was written in a style understandable to patients while keeping it concise. The first draft by MG was read by several co‐authors (CS, SF, KM, and SW) who all have extensive experience in cancer care and complementary medicine.

In the next step, the draft was tested on 15 patients to check its content and scope. It was distributed as a simple A4 printout and as an online document (via computer). No further adaptations resulted from this test.

For the final study, the text was printed as a brochure in a compact format. The final printed version included 13 pages in A5 format. (The brochure is attached as eSupplement 1).

The participants were briefly instructed in the various centers by means of a cover letter and then carried out the intervention independently. The authors were available for questions. Answering the questions and reading the decision aid took place consecutively. There were no regulations regarding the location where the study was carried out.

### 2.2 Questionnaire

In parallel, we developed a pre‐post cross‐sectional survey with a questionnaire consisting of 28 questions in two parts. The first part was answered before reading the decision aid, and the second part afterwards (questions 1–14 and 15–28, respectively).

The questionnaire was divided into five sections:

- Demographics (overview of gender, age, marital status, educational attainment, and socioeconomic status)

- Knowledge questions (these were more specific to the topic of homeopathy and were designed to evaluate knowledge before and after reading the decision aid, with the same three questions asked before and after reading; each answer option was numerically coded, “I don't know‐answers” were not included in analysis)
- General use of homeopathy (previous use of homeopathy)
- Use of homeopathy for specific medical conditions (table with six medical conditions. Patients were asked to rate on a Likert scale their likelihood of using homeopathy for each condition before and after reading)
- Evaluation of the decision aid (opportunity to rate the information they have read; comments could also be made at the end)

Most questions were closed questions, some of them multiple choice. A few questions also offered an open answer field (question 9 and additional comments)—however, there was no statistical evaluation of these answers, the answers served to provide a supplementary perspective. Due to the low response rate, no evaluation could take place. (The questionnaire is attached as eSupplement 2).

The questionnaire was tested on 15 individuals who had previously reviewed decision aids. Adjustments were made in the areas of demographics, general use of homeopathy, and specific medical conditions. In this study, specific question formulations were modified, and unnecessary questions were eliminated following the review process to reduce the survey's length. For example, in question 10 (How much money would you be willing to spend on homeopathy?), patients mentioned a need for a time frame (per month). The pre‐test phase was intended solely for qualitative assessment of content, clarity, and comprehensibility, not for statistical evaluation of the questionnaire.

### 2.3 Participants

From April 2022 to December 2022, we recruited cancer patients who were willing to participate in the study through multiple centers. A total of 200 brochures, including decision‐making aids were sent to the various centers. Of these, 123 were completed, returned, and evaluated. Included in this were rehabilitation clinics, hospitals, specialized medical practices, and cancer societies. All participating centers were located in Germany and had a focus on oncology. Participation required a past or ongoing cancer treatment. There were no age restrictions. All participants were informed in advance participation was voluntary.

### 2.4 Tests for Normality

To assess whether the data followed a normal distribution, both the Kolmogorov–Smirnov and Shapiro–Wilk tests were conducted.

### 2.5 Wilcoxon Signed Rank Test

This test was used to determine whether participants' opinions on the use of homeopathy for specific medical conditions changed after reading a decision aid.

### 2.6 McNemar's Test and Wilcoxon Signed Rank Test for Knowledge Assessment

Knowledge improvement was assessed using McNemar's test (for categorical variables) and the Wilcoxon Signed Rank test (for continuous variables).

### 2.7 Mann–Whitney *U* Test

This test was applied to examine differences in decision‐making based on prior homeopathy use and gender differences.

### 2.8 Spearman's Rho Correlation Analysis

The relationship between socioeconomic status and changes in knowledge or attitudes was explored using Spearman's rank correlation coefficient.

### 2.9 Kruskal–Wallis Test

This test was used to examine whether knowledge changes differed by educational background and school qualification.

### 2.10 Ordinal Regression Analysis (Nagelkerke R^2^)

This analysis was conducted to evaluate the impact of question 7 on the knowledge questions.

### 2.11 Informed Consent

Informed consent was given by all participants by filling in the questionnaire. This study followed the Institutional Research Ethics Board and the Declaration of Helsinki guidelines.

### 2.12 Ethical Approval

The survey was approved in advance by the Ethics Committee of the “Universitätsklinikum Jena” (Reg. No.: 2022‐2510‐Bef). The basis for this was the anonymous survey of the participants and the preservation of all data protection‐relevant aspects. In particular, the authors paid attention to the vulnerable situation of the patients, and no one was urged to participate. No participant received an expense allowance.

## 3 Statistics

IBM SPSS Statistics for Mac, Version 24.0 was used for data collection and the statistical analysis. For the statistical analysis, the five sections mentioned above were used. Demographic data and evaluation were analyzed purely descriptively through counting and subsequent graphical representation. The knowledge questions and medical conditions sections were used to examine knowledge acquisition and decision‐making behavior before and after reading the decision aid.

The statistical methods used in this study are mentioned and described in chapters 5.4–5.9. Overall, the study employed non‐parametric statistical tests due to non‐normally distributed data and used a range of methods to assess knowledge, opinion changes, and influencing factors. The findings emphasize the need for targeted communication strategies to improve health literacy and support informed decision‐making regarding homeopathy.

Only those datasets with complete responses both before and after reading the decision aid were included in the statistical analysis. For reasons of transparency, we have also given the total N in Figures 2 and 3 in absolute numbers of before and after.

## 4 Results

### 4.1 Demographics

In total, 123 participants took part in the survey. Among them, 31 were male, and 85 were female. The average age was 58 years, with the oldest participant being 80 and the youngest being 26. Additional demographic information can be found in Table 1.

**Table 1:** Demographic data.

Variable	*N*	%
**Gender**		
Male	31	25,20
Female	85	69,10
No answer	7	5,70
**Age**		
Under 30	3	2,40
31–50	18	14,60
51–70	79	64,20
71–80	15	12,20
No answer	9	7,30
**Marital status**		
Single	15	12,20
Married	84	68,30
Widowed	7	5,70
Divorced	10	8,10
No answer	7	5,70
**Graduation**		
High	50	40,70
Middle	29	23,60
Low	34	27,60
No answer	10	8,10
**Educational qualification**		
Vocational training	79	64,20
Degree	33	26,80
No answer	11	8,90
**Socioeconomic status**		
Low (1–3)	11	8,90
Middle (4–6)	67	54,50
High (7–10)	37	30,10
No answer	8	6,50

*Note:* Table 1 presents the demographic data of the sample (*N* = 123). The data include information on gender, age, marital status, educational attainment, educational qualification, and socioeconomic status.

Educational qualifications were categorized as follows:

High: High School (Abitur/Polytechnische Oberschule)

Middle: Secondary School (Mittlerer Abschluss)

Low: Secondary School (Haupt‐(Volks‐)schulabschluss)

Socioeconomic status was self‐rated using the MacArthur Scale, where 1 = very low and 10 = very high.

### 4.2 Tests of Normality (Kolmogorov–Smirnov and Shapiro–Wilk Tests)

Both the Kolmogorov–Smirnov and Shapiro–Wilk tests indicate significant deviations from normality for all variables (*p* < 0.001). Since the data are not normally distributed, non‐parametric statistical tests were used for further analysis.

### 4.3 Use of Homeopathy for Specific Medical Conditions (Wilcoxon Signed Rank Tests)

**Figure 1:**
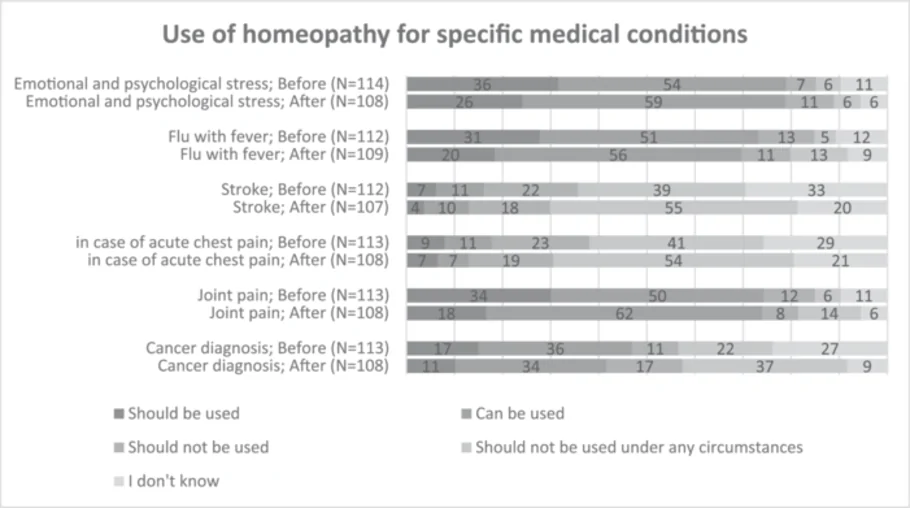
“Use of homeopathy for specific medical conditions.” Using a Likert scale, participants were surveyed before and after reading about the extent to which they would use homeopathy for specific medical conditions. The results are presented in the above table in absolute numbers.

No significant change in willingness to use homeopathy was found for *psychological and emotional stress* (*p* = 0.225, r = 0.12) or for *acute chest pain* (*p* = 0.058, r = 0.22). The corresponding effect sizes indicate small effects. In contrast, significant changes after the use of the decision aid were observed for several other conditions. Significant differences were found for *cold with fever* (*p* = 0.006, r = 0.29), *stroke* (*p* = 0.001, r = 0.39), *joint complaints* (*p* = 0.001, r = 0.33), and *cancer* (*p* = 0.004, r = 0.32). The effect sizes ranged from small to medium, with the largest effect observed for *stroke*. After applying a Bonferroni correction for multiple testing, the changes for *cold with fever*, *stroke*, *joint complaints*, and *cancer* remained statistically significant, whereas the results for *psychological and emotional stress* and *acute chest pain* remained non‐significant. Overall, the findings suggest that the decision aid influenced willingness to use homeopathy differently depending on the medical condition, with significant changes and small to medium effect sizes particularly observed for more severe or potentially life‐threatening conditions (see Table 2 and Figure 1).

**Table 2:** Changes in willingness to use Homeopathy for specific medical conditions before and after the Decision Aid (Wilcoxon Signed‐Rank Tests).

Condition	*p*‐value	Interpretation	Valid answers (total *N*)	Standard error	Bonferroni	Effect Size (r)
Psychological and emotional stress	0.225	No significant change	96	37,515	1.35	0.124
Cold with fever	0.006	Significant change	93	34,460	0.036	0.285
Stroke	0.001	Significant change	72	21,030	0.006	0.388
Acute chest pain	0.058	No Significant change	76	27,973	0.348	0.217
Joint complaints	0.001	Significant change	97	42,997	0.006	0.334
Cancer	0.004	Significant change	82	35,176	0.024	0.318

Abbreviations: *N*, Number of responses included in the evaluation; *p*‐value, Upper‐tail probability of the test statistic assuming that the null hypothesis is true; r, measures the strength of the difference between two connected samples, interpreting values from 0.1 (small), 0.3 (medium) to 0.5 (strong) according to Cohen.

### 4.4 Placebo, Potentization, and Side Effects Knowledge (Wilcoxon Signed Rank Tests and McNemar Change Test)

Changes in participants' knowledge about placebo effects, potentization, and side effects of homeopathy were assessed before and after reading the decision aid.

Knowledge about placebo effects increased significantly following the intervention (Wilcoxon signed‐rank test, *p* = 0.001, *N* = 110), with a medium effect size (*r* ≈ 0.31).

Similarly, knowledge about potentization showed a significant improvement (McNemar test, χ^2^ = 14.694, *p* = 0.001, *N* = 123). The effect size was in the medium range (φ ≈ 0.35).

**Figure 2:**
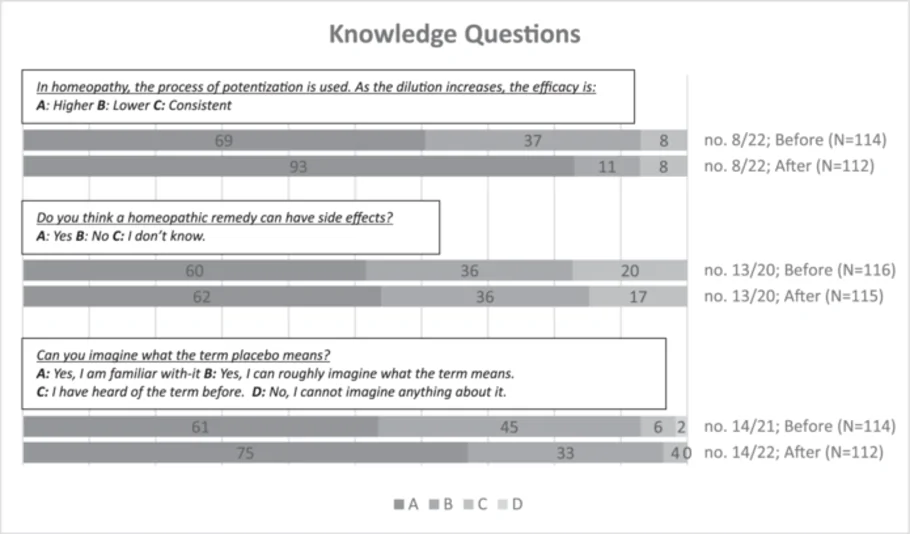
“Knowledge questions.” Here is the distribution of the knowledge questions (3 in total) presented in absolute numbers. The corresponding questions were included with answer options for clarity.

In contrast, knowledge about side effects of homeopathy did not change significantly between pre‐ and post‐measurement (McNemar test, χ^2^ = 0.643, *p* = 0.424, *N* = 88), and the effect size was small (φ ≈ 0.09). Overall, the decision aid was associated with meaningful knowledge gains regarding placebo effects and potentization, but not regarding side effects (see Table 3 and Figure 2).

**Table 3:** Changes in knowledge about placebo effects, potentization, and side effects of homeopathy before and after the decision aid.

Variable	Test used	*p*‐value	Interpretation	Valid answers (total *N*)	Standard error	Chi‐square	Bonferroni	Effect size (r/φ)
Placebo knowledge	Wilcoxon Signed Rank	0.001	Significant improvement	110	36.971		0.006	0.31 (r)
Potentization knowledge	McNemar Change Test	0.001	Significant improvement	123		14.694	0.006	0.35 (φ)
Side effects knowledge	McNemar Change Test	0.424	No significant change	88		0.643	2.544	0.085 (φ)

Abbreviations: φ (Phi coefficient), the interpretation is analogous to other effect sizes (according to Cohen); N, Number of responses included in the evaluation; *p*‐value, Upper‐tail probability of the test statistic assuming that the null hypothesis is true; r (coefficient), measures the strength of the difference between two connected samples, interpreting values from 0.1 (small), 0.3 (medium) to 0.5 (strong) according to Cohen.

### 4.5 Influence of Prior Homeopathy Use on Decision‐Making (Mann–Whitney *U* Test)

Prior users of homeopathy made different decisions after reading the decision aid compared to non‐users. The response behavior of the participants was assessed in relation to the previous use of homeopathy and the use of it after reading the decision aid (question 9/19). There was a significant difference between participants who previously used homeopathy and those who did not (*N* = 114; SD = 170.525; *p* < 0.001).

The effectiveness of decision aids may depend on prior experiences and beliefs, requiring tailored interventions.

### 4.6 Correlation Analysis (Spearman's Rho)

This analysis investigates whether socioeconomic status is associated with changes in participants' knowledge or opinions. For this purpose, the influence of socioeconomic status on decision‐making behavior in the topics “knowledge questions” and “specific medical conditions” was evaluated. No significant correlations were found, indicating that socioeconomic status does not influence participants' changes in knowledge or attitudes toward homeopathy. Educational interventions on homeopathy may be equally effective across different socioeconomic groups (see Table 4).

**Table 4:** Correlation between socioeconomic status and changes in knowledge and attitudes toward Homeopathy (Spearman's Rho).

Variable (difference)	Spearman's rho (rs)	*p*‐value	Interpretation	Valid answers (total *N*)	Bonferroni
Socioeconomic Status & Side Effects Knowledge	0.075	0.488	No correlation	87	2.928
Socioeconomic Status & Potentization Knowledge	−0.007	0.945	No correlation	115	5.67
Socioeconomic Status & Placebo Knowledge	0.074	0.446	No correlation	109	2.676
Socioeconomic Status & Change in Opinion on Medical Conditions	−0.013	0.899	No correlation	102	5.394

Abbreviations: *N*, Number of responses included in the evaluation; *p*‐value, Upper‐tail probability of the test statistic assuming that the null hypothesis is true; R^2^, statistical measure used to evaluate fit quality for logistic regression models; Spearman's rho (rs), nonparametric rank correlation coefficient, which measures the strength and direction of monotonic relationships between two variables (Cohen).

### 4.7 Group Differences by Education and School Qualification (Kruskal–Wallis Tests)

This analysis tests whether changes in knowledge or attitudes differ based on educational background. No significant differences were found based on education level or school qualification. Knowledge changes do not depend on formal education, suggesting that decision aids may be equally effective across different educational backgrounds (see Table 5).

**Table 5:** Differences in knowledge gains by educational background and school qualification (Kruskal–Wallis Tests).

Grouping factor	Variable (difference)	*p*‐value	H	Interpretation	Valid answers (total *N*)	Bonferroni	η^2^
Educational level	Opinion on medical conditions	0.982	0.006	No difference	100	5.892	−0.0101
	Side effects Knowledge	0.822	0.393	No difference	86	4.932	−0.0072
	Potentization Knowledge	0.185	1.757	No difference	112	1.11	0.007
	Placebo Knowledge	0.575	0.314	No difference	106	3.45	−0.0066
Highest school qualification	Opinion on medical conditions	0.431	2.756	No difference	101	2.586	−0.0025
	Side effects Knowledge	0.884	0.652	No difference	85	5.304	−0.029
	Potentization Knowledge	0.816	0.94	No difference	113	4896	−0.0189
	Placebo Knowledge	0.957	0.316	No difference	107	5742	−0.026

Abbreviations: η^2^ (Eta), a measure of effect size that represents the proportion of the explained variance in the rankings of the dependent variable; H, Test statistics for the Kruskal‐Wallis test; N, Number of responses included in the evaluation; *p*‐value, Upper‐tail probability of the test statistic assuming that the null hypothesis is true; R^2^, statistical measure used to evaluate fit quality for logistic regression models.

### 4.8 Influence of Prior Knowledge From the Patients (Ordinal Regression Analysis)

Regression analyzes were conducted to examine whether prior knowledge about homeopathy influenced the knowledge gains achieved through the decision aid. Topic‐specific prior knowledge served as the predictor, and post‐intervention knowledge gain as the outcome variable. For knowledge regarding side effects of homeopathic treatments, no significant association between prior knowledge and knowledge gain was found (*p* = 0.735). The model explained none of the variance (Nagelkerke R^2^ = 0.000), indicating that knowledge improvement in this domain occurred independently of participants' baseline knowledge.

In contrast, lower prior knowledge significantly predicted knowledge gains related to the procedure of potentization (*p* = 0.024). However, the amount of explained variance was small (Nagelkerke R^2^ = 0.056), indicating a small effect size. Similarly, lower prior knowledge was a significant predictor of increased understanding of the placebo concept (*p* = 0.012). The model accounted for 7.2% of the variance in knowledge gain (Nagelkerke R^2^ = 0.072), which also represents a small effect. Overall, the findings suggest that the decision aid improved knowledge about side effects regardless of participants' prior knowledge. For potentization and the placebo concept, participants with lower baseline knowledge showed slightly larger knowledge gains, although the overall effects were small (see Table 6).

**Table 6:** Influence of prior knowledge about homeopathy on post‐intervention knowledge gains (Ordinal Regression‐analysis).

Queried knowledge parameter	*p*‐value	Significance	Nagelkerke R^2^	Valid answers (total *N*)	Bonferroni
Side effects of homeopathy	0.735	Not significant	0.000	88	4.41
Procedure of the Potentiation	0.024	Significant	0.056	116	0.144
Placebo term	0.012	Significant	0.072	110	0.06

Abbreviations: *N*, Number of responses included in the evaluation; *p*‐value, Upper‐tail probability of the test statistic assuming that the null hypothesis is true; R^2^, statistical measure used to evaluate fit quality for logistic regression models.

### 4.9 Gender Differences (Mann–Whitney *U* Test)

Furthermore, the response behavior in relation to the gender of the participants was examined.

No statistically significant differences were found about changes in the assessment of medical conditions (*p* = 0.574), knowledge gains related to the placebo concept (*p* = 0.782), or knowledge about potentization (*p* = 0.600). The associated effect sizes were consistently very small (*r* = 0.03–0.06) and therefore practically negligible.

A significant gender difference emerged only for knowledge gains concerning the side effects of homeopathic remedies (*p* = 0.009). The effect size was in the small to lower medium range (*r* ≈ 0.28), indicating that gender had a noticeable, though overall modest, influence on knowledge acquisition in this domain.

In the knowledge question regarding side effects, it was observed that the correctness of the answer of the female participants increased (mean = 0.147) whilst male participants' correctness decreased (mean = −0.2500). Men were less likely to correctly answer questions about side effects after the intervention, while women showed improved accuracy. Future decision aids may need targeted messaging to ensure both genders equally benefit from the provided information (see Table 7).

**Table 7:** Gender Differences in knowledge gains after reading the decision aid (Mann‐Whitney *U* Test).

Variable (difference)	*p*‐value	Interpretation	Valid answers (total *N*)	Bonferroni	Effect Size (r)
Opinion on medical conditions	0.574	No difference	103	3.444	0.055
Side effects Knowledge	0.009	Significant gender difference	88	0,054	0.278
Potentization Knowledge	0.600	No difference	116	3.6	0.048
Placebo Knowledge	0.782	No difference	110	4.692	0.027

Abbreviations: *N*, Number of responses included in the evaluation; *p*‐value, Upper‐tail probability of the test statistic assuming that the null hypothesis is true; r, measures the strength of the difference between two connected samples, interpreting values from 0.1 (small), 0.3 (medium) to 0.5 (strong) according to Cohen.

### 4.10 Evaluation of Decision Aid

Two‐thirds of respondents (66.1%) indicated that they felt well informed after reading the decision aid (22.6% “totally agree”; 43.5% “applies rather”). Similarly, 61.7% reported that their knowledge on the topic had expanded (25.2% “totally agree”; 36.2% “applies rather”). Approximately 17% stated that their opinion had changed after reading the decision aid. For 23.7% (9.7% “totally agree” and 14% “applies rather”) homeopathy remained a treatment option.

The clarity and illustrations were mostly rated good. The neutrality and presentation of scientific findings were positively evaluated with 32.5% “totally agree” and 35.2% “applies rather” for each category.

In question 18, patients were asked if there were any new pieces of information, they found interesting. The manufacturing process and scientific studies were each selected 56 times, while the approval process was chosen 46 times.

Regarding the amount of information provided, 71.4% rated it as just right.

**Figure 3:**
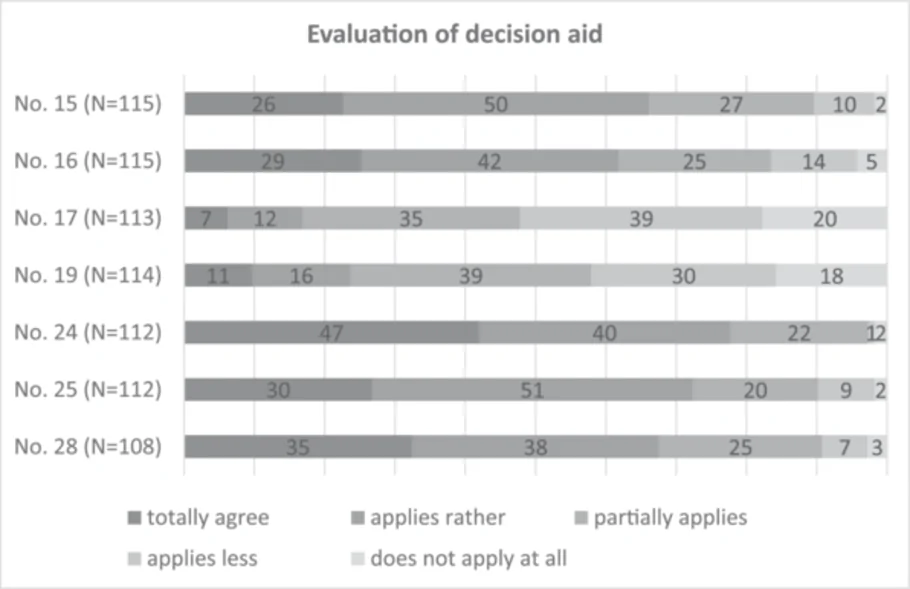
“Evaluation of decision aid.” This overview shows the evaluation of the decision aid. Participants were surveyed using a Likert scale, and the results are presented in absolute numbers. No. 15 *I feel well informed about homeopathy through the decision‐making aid*. No. 16 *I was able to expand my knowledge about homeopathy through the decision‐making aid*. No. 17 *The decision‐making aid has changed my opinion on homeopathy*. No. 19 *After reading the decision‐making aid, a homeopathic therapy is an option for me*. No. 24 *The brochure was formulated in a way that I could understand*. No. 25 *The illustrations in the brochure helped me understand better*. No. 28 *The decision‐making aid presents scientific findings neutrally and provides an overview of homeopathy*.

Furthermore, 81.8% of respondents stated that they would recommend the decision aid to others. The results are presented in Figure 3.

### 4.11 Analysis of Open Questions and Comments

In question 9.2, participants were asked to indicate in an open‐ended question what they had used homeopathy for in the past. In Table 8, the answers were sorted and listed. Furthermore, an overview of the participants' comments at the end of the questionnaire was prepared (Table 9).

**Table 8:** Overview of what patients have used homeopathy for in the past (Question 9.2).

Category/Indication	Response
Gastrointestinal issues	Stomach problems, nausea, reflux, bladder infections, abdominal pain after antibiotics
Respiratory infections & cold symptoms	Cough, cold, flu, sinusitis, long‐lasting cough, bronchitis, children's colds
Pain & injuries	Joint pain, back pain, tennis elbow, bruises, sports injuries, post‐surgery tendon/ligament care, trauma, wounds
Fever & common illnesses	Fever, children's illnesses (e.g., measles, chickenpox), influenza
Migraines/headaches	Migraine, headaches, tension
Stress/psychological conditions	Nervousness, anxiety, stress, inner restlessness, sleep disturbances, emotional support
Women's health	Menopause, gynecological issues, fertility support
Cancer‐related/therapy support	Side effects of cancer treatment (nausea, fatigue, immune support, reduction of conventional medication burden)
Skin/mucous conditions	Neurodermatitis, psoriasis, eczema, warts, furuncles, wounds, dry mucous membranes, varicose veins
Dental/ENT issues	Tooth operations, ear infections, eye issues (Euphrasia), sinusitis
Immune support	Strengthening immunity, recovery support, neuropathy relief, general health maintenance
Homeopathy for children	Falls, minor injuries, fever, colds, preventive measures
Animal use	Pain and nausea management for pets
Other/general use	“Used for everything,” miscellaneous minor ailments, general physical, psychological, and emotional support

Abbreviation: ENT, Ear, Nose, and Throat.

**Table 9:** Summary of feedback on the homeopathy decision aid (survey responses).

Theme	Key points/Examples
Perceived clarity and understanding	Some questions were confusing, incomplete, or leading (e.g., Q6, Q4, Q5, Q8, Q12). Participants suggested simpler, clearer wording to avoid misunderstanding.
Neutrality versus bias	Several respondents felt the decision aid was biased against homeopathy, despite aiming for neutrality. Some suggested including positive aspects or experiences.
Homeopathy versus conventional medicine	Many emphasized that homeopathy should not replace conventional treatment (e.g., chemotherapy) but could be used complementarily. Some noted that it works well in combination with standard medicine.
Belief and subjective experience	Participants mentioned the role of belief (“faith moves mountains”) and personal experiences, e.g., successful outcomes with Arnica, Feigenbaum therapy, symptom relief, and scar healing.
Placebo effect	Multiple comments highlighted that the placebo effect plays a role in perceived benefits, even in conventional medicine. Some suggested this should be acknowledged more clearly, including positive psychological effects.
Scope and content	Requests for additional information: how to choose the right remedy, effects on children and animals, background of homeopathy founders, production methods, and “subtle energy” principles. Some felt the brochure was too focused on scientific critique and ignored practical or historical context.
Presentation/title	Some found the title confusing without prior knowledge; suggested including context or more background about the organization behind the aid.
General attitudes toward homeopathy	Responses varied: some participants would use it only as a supplement, some had mixed experiences, some continue to believe strongly, and some consider it ineffective. A few suggested broader disseminations of information to the public.
Survey logistics	Participants noted that allowing external references during the survey may bias results; recommended independent completion.
Suggestions for improvement	Include both positive and negative aspects, clarify questions, emphasize placebo and psychological effects, give practical guidance on remedy selection, consider patient experience and subjective outcomes, and distinguish clearly between homeopathy and other complementary medicine.

Abbreviation: Q, questions from survey.

## 5 Discussion

### 5.1 Impact of the Decision Aid on Knowledge and Perception

To our knowledge, this is the first study to investigate the impact of a patient decision aid on perceptions and knowledge about homeopathy. The objective of this survey study was to examine the impact of our decision aid on the topic of homeopathy.

As for the quality of the decision aid, we were able to show that adherence to the criteria of the IPDAS is feasible even for a topic related to alternative medicine [13]. Clarity and presentation of evidence were particularly important to us [14]. Furthermore, through a pre‐test, we tried to eliminate potential sources of error and considered the feedback from participants. The use of a print version was intended to enhance participation through a user‐friendly design [15].

Overall, the decision aid expanded participants' knowledge and encouraged more differentiated reflection on homeopathy, particularly concerning medical conditions. Evaluation responses confirmed general acceptance. The decision aid's neutrality and representation of scientific evidence were rated positively. 81.8% of participants reported they would recommend it. Patients generally felt well‐informed and gained new insights. Comprehensibility and use of illustrations received mostly positive feedback.

Ordinal regression analyzes indicated that participants with lower prior knowledge benefited more from the decision aid, particularly in understanding the concepts of “potentiation” and the “placebo difference,” considering that only 5.6% of the predictor's explanation of variance is contributed. However, no improvement was found regarding knowledge of side effects. These findings suggest the decision aid may support knowledge acquisition among less pre‐informed individuals.

An important consideration emerged regarding whether scientifically implausible claims, such as the notion that the effect of homeopathy increases with higher dilution (potentiation), should be explicitly included in patient decision aids. Despite the lack of empirical support for this concept, it was included in our material because it represents a core tenet of homeopathy and is widely circulated in public discourse. Omitting such a statement might have been interpreted by users as a lack of neutrality or as disregarding the beliefs of homeopathic proponents, potentially undermining the credibility of the decision aid. Nonetheless, from a clinical perspective, future adaptations of the decision aid for use in practice should consider framing such claims more cautiously, clearly labeling them as originating from the homeopathic doctrine and contrasting them with the scientific consensus. This would help balance transparency with scientific rigor while maintaining user trust.

### 5.2 Barriers to Changing Attitudes or Behaviors

Despite these knowledge gains, we observed limited changes in participants' attitudes. Perceptions of homeopathy remained relatively stable (34.5% applies rather; 17.7% does not apply at all). This could be due to participants' pre‐existing opinions, and it's possible that the concise format of the decision aid might have limited the extent of change in this aspect [16].

Participants with prior experience using homeopathy (*N* = 58) continued to view it as a therapeutic option (totally agree=11 applies rather = 11 partially applies = 24). Even among those without prior use (*N* = 56), some indicated openness to future use (partially applies = 15 applies less = 21 does not apply at all = 15).

Interestingly, around 80% of participants reported familiarity with the topic, yet this was not reflected in their answers to the knowledge questions. This suggests a potential overestimation of knowledge.

### 5.3 Role of Prior Beliefs, Placebo Effects, and Emotional Needs

Why, nonetheless, does the attitude of the study participants not change? Even with awareness of uncertain or unproven efficacy, participants might value the placebo effect, which can be perceived as beneficial [17].

The perception of homeopathy as harmless may further support its use, as it is seen as a low‐risk intervention. Certainly, the context of our study should be considered here. Cancer therapy represents an exceptional situation for every individual. Fried et al. observed in 2003 that patients, in the face of increasing severity of an illness, are also willing to accept potential failures [18].

Furthermore, often perceived as personalized and attentive, possibly fulfilling emotional and psychological needs unmet by conventional medicine [19]. This may explain why a decision aid in isolation has a limited impact when prior experiences or social influences reinforce positive perceptions. Combining it with an informative discussion could enhance the effect, integrating the decision aid as a tool into the conversation [20].

It is evident from our data that some participants who reported using homeopathy for stress‐related psychological and emotional factors indicated continued willingness to use it after reading the decision aid. This aligns with the available literature, which demonstrates that homeopathy is often used in the treatment of psychological disorders but also showed no effect in this context [21]. It may indicate that contextual and relational factors play a role in perceived benefit in this context, as emotional and psychological distress is heavily influenced by subjective experiences.

More concerning is that some participants still endorsed the use of homeopathy for acute physical conditions (e.g., fever, stroke, chest pain, joint pain, and cancer), even after reading the decision aid and recognizing its lack of efficacy—either categorizing them as “can be used” or expressing the belief that homeopathic methods “should be used” in these situations. This could be related to limited health literacy, which has been described in parts of the German population and may be more prevalent among older adults in oncology settings [22, 23].

### 5.4 Implications for Improving Health Literacy and Patient Communication

Improving health literacy, particularly among older populations, is essential for enabling informed decisions. Participants may have difficulty understanding provided health information and apply it to make decisions regarding health matters, or incorporating it into their decision‐making process [24].

Although participants may understand the lack of efficacy after reviewing the decision aid, behavioral or attitudinal change does not necessarily follow. This is because personal experiences, emotional attachments, or even the social environment may support their existing stance.

Prior surveys indicate that positive personal experience is a key driver of alternative medicine use [25, 26]. In case of homeopathy, commonly used for diseases with spontaneous improvement such as common cold or diseases in which a placebo‐effect may help, positive experiences may lead to generalization of its perceived effectiveness, even in serious diseases. In emotionally distressing situations such as cancer diagnosis, patients may revert to previously perceived successful behavioral strategies. This may also reflect unmet emotional or spiritual needs within modern health care systems, as some patients look for more comprehensive approaches to their disease. Integrating spiritual care could support decision‐making in emotionally complex situations.

Embedding decision aids within physician‐patient interactions, where questions and concerns can be addressed in a supportive environment, may increase their utility. Furthermore, promoting evidence‐based complementary therapies, such as phytotherapy or mind‐body interventions, within integrative oncology could bridge the gap between conventional and alternative care.

The role of integrative oncology should also be further explored, as it may provide a bridge between conventional and alternative medicine [27]. By incorporating scientifically validated complementary therapies, such as phytotherapy [28] and mind‐body interventions, into mainstream cancer care, patients may receive holistic treatment options that align with their beliefs while maintaining an evidence‐based approach. Furthermore, addressing the spiritual needs of patients within healthcare systems could enhance patient satisfaction and trust, ultimately influencing decision‐making in a way that prioritizes both emotional well‐being and scientific validity. Future research should explore how personal experiences shape health attitudes and develop strategies that support evidence‐based decisions while respecting individual values.

Homeopathy is considered part of complementary and alternative medicine. These therapy forms include healing methods such as yoga, meditation, dietary supplementation, traditional Chinese medicine, phytotherapy, and many more.

However, it is important to distinguish that some of these therapy options are supported by varying levels of evidence or promising data (e.g., for certain plant‐derived active ingredients), in contrast to homeopathy.

## 6 Limitations

The most important limitation of this single‐arm, pre‐post observational study is the low number of participants, which, however, could not have been realized differently due to the time constraints of the survey. With a larger number of participants, it would have been possible to better identify the factors influencing decision‐making. The fact that the study included patients who may have been biased cannot be ruled out, especially the fact of a selection bias because no randomization was made. In this context, it must be emphasized that most participants were recruited from conventional medical centers. This recruitment strategy constitutes a significant selection bias, as patients treated in institutions practicing evidence‐based medicine represent a specific subgroup and may not be representative of the broader patient population. The way of interviewing patients from institutions practicing evidenced base medicine is per se a selection of a special type of patients. Additionally, it is worth noting that due to the simultaneous reading of the decision aid and answering the questionnaire, it is inevitable to have a certain bias towards what was read.

Since the survey of the study participants only took place within a time limit, confounding variables cannot be ruled out. There is a risk that participants will adjust their answers subjectively or due to social desirability, which can affect the validity of the data. Moreover, the lack of data on important clinical variables such as cancer stage, time since diagnosis, and active treatment status severely limits the interpretation of the results, as these factors are likely to have a substantial impact on therapeutic decision‐making.

Also, the gender distribution in this study is important. The proportion of female participants was relatively high, and a more homogeneous gender distribution would have been desirable. This imbalance requires a more cautious interpretation of the findings, particularly regarding gender‐based analyzes. Observed gender differences in knowledge outcomes should be interpreted with caution due to the sample size and should be considered hypothesis‐generating rather than confirmatory. Furthermore, we did not inquire about the stage of cancer, which undoubtedly also has an impact on the choice of therapy, in the same way as the fact that we conducted the survey in a conventional medical setting.

Furthermore, it is critical to see that the “I don't know” answers in the survey can be associated with uncertainty, instead of taking a position between the “yes” or “no” answers.

## 7 Conclusion

The provision of comprehensive and understandable information for patients plays a crucial role in healthcare. Good patient information is essential not only for understanding one's own health but also for enabling patients to actively participate in their own care. Numerous studies have already demonstrated that decision aids can play a role in the decision‐making process [12]. In the context of the growing significance of patient satisfaction, it is crucial to prepare patients for decisions using evidence‐based information. Doctor‐patient interaction, as well as education and information, are among the most frequently mentioned issues in patient satisfaction surveys [29]. As own experience and the human interaction is important especially in decision‐making in situations of uncertainty, also well‐designed information material does not replace the patient‐physician interaction and in case evidence is against patients' former own experience and the socially prevailing attitudes, this interaction gets an even higher importance.

Continuous evaluation and adaptation of information strategies in collaboration with patients are crucial to ensuring that patients can make informed decisions about their health. Only through effective communication can patients truly be empowered to actively participate in their own healthcare.

To translate these findings into clinical practice, oncology teams should consider integrating decision aids as structured components within the patient consultation process. This integration could occur during shared decision‐making encounters, where decision aids serve as a neutral foundation for discussion, helping patients articulate their values and questions. Facilitating this process may require training healthcare professionals in the effective use of such tools and ensuring sufficient consultation time for individualized guidance.

Moreover, to address the varying levels of health literacy among patients—particularly in older populations or those facing the psychological burden of a cancer diagnosis—decision aids should be adapted to different comprehension levels. This may include the use of simplified language, visual support, or multilingual versions that account for cultural contexts and communication preferences. Co‐developing materials with patient representatives can enhance both clarity and relevance.

Ultimately, embedding decision aids into routine oncological care not only supports informed choices but also reinforces the therapeutic alliance between patients and clinicians. This approach aligns with the principles of person‐centered care and contributes to improved satisfaction, trust, and adherence, especially in emotionally complex decision‐making contexts.

## Author Contributions

**Maximilian Gimbel:** conceptualization, methodology, writing – review and editing, software, validation, formal analysis, writing – original draft, visualization. **J. Hübner:** conceptualization, methodology, writing – review and editing, supervision, project administration, investigation, resources, data curation. **C. Stoll, St. Fuxius, F.J. Prott, K. Münstedt, B. Zomorodbakhsch, Sandra Wittmann,** and **Karin Kastrati:** investigation, resources, data curation. **J. Hübner:** conceptualization, methodology, writing – review and editing, supervision, project administration, investigation, resources, data curation.

## Ethics Statement

The study was approved by the ethic committee of the university hospital Jena (vote No: 2022‐2510‐Bef).

## Conflicts of Interest

The authors declare no conflicts of interest.

## Patient or Public Contribution

All authors, caregivers, and survey centers involved have extensive experience in cancer therapy. Patients and caregivers were integral to our study on the effectiveness of a decision‐making aid for homeopathy in cancer therapy. Subsequently, participants were asked to read the decision‐making aid and complete a questionnaire both before and after its use, providing valuable data on its impact on their knowledge and decision‐making confidence. The responses from these questionnaires were analyzed to assess the aid's effectiveness. Their contributions ensured that the study was grounded in real‐world relevance and utility.

## Transparency Statement

The lead author Maximilian Gimbel affirms that this manuscript is an honest, accurate, and transparent account of the study being reported; that no important aspects of the study have been omitted; and that any discrepancies from the study as planned (and, if relevant, registered) have been explained.

## Supporting information


Supporting File 1

Supporting File 2

## Data Availability

The data that support the findings of this study are available from the corresponding author upon reasonable request.
