# Supplementary material for: Efficacy of a Decision‐Making Aid About Homeopathy in Patients With Cancer: A Single‐Arm, Pre–Post Observational Study
Source: Health Sci Rep. 2026 Jul 30;9(8):e72943. doi: 10.1002/hsr2.72943 (PMC13420187; doi:10.1002/hsr2.72943)
Supplement: Supplementary file 1 — Supporting File 1 [file HSR2-9-e72943-s001.docx]

*
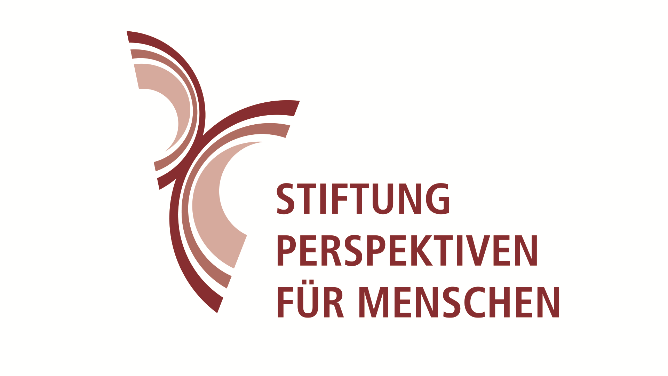
*

*
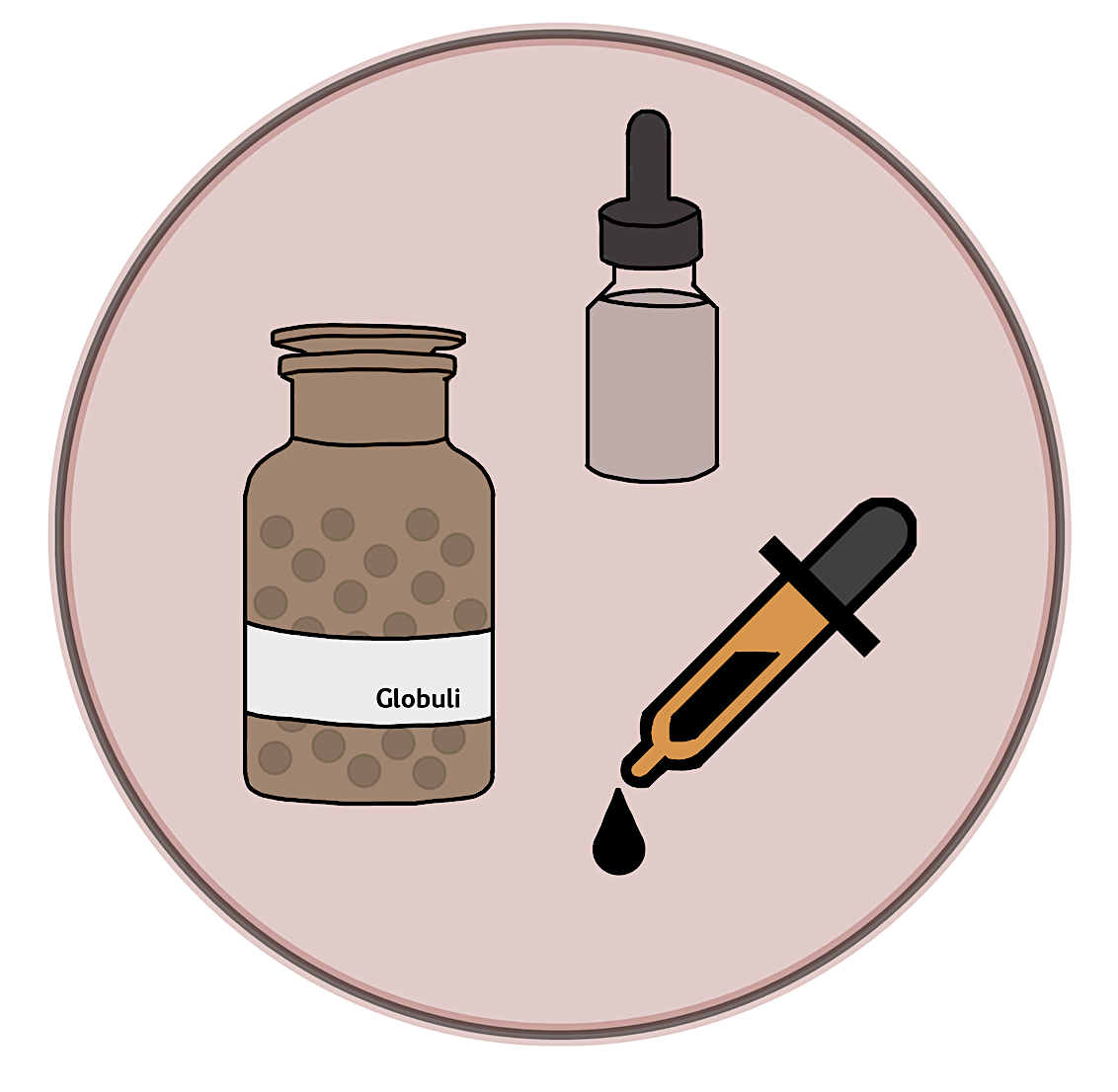
*

Entscheidungshilfe Homöopathie

**Was ist Homöopathie?[1]**

Eine eckige Klammer mit einer Zahl zeigt an, dass hier eine Quelle verwendet wurde. Diese finden Sie auf Seite 11 und 12.


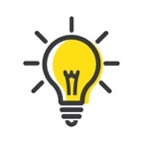


Die Homöopathie wurde vor mehr als 200 Jahren von dem deutschen Arzt Samuel Hahnemann entwickelt. Sie geht von zwei wesentlichen Annahmen aus:

- Homöopathie nutzt Substanzen die beim Gesunden ähnliche Symptome hervorrufen wie die zu behandelnde Krankheit. 🡪 **Gleiches heilt Gleiches**
- Die Substanzen werden schrittweise verdünnt. Je höher der Grad der Verdünnung, desto höher soll die Wirkung sein. Diese „Verstärkung“, soll durch das Verschütteln der Substanz in der Lösung erfolgen. 🡪 **Potenzierung**

Homöopathische Arzneimittel werden aus verschiedenen Materialien hergestellt. Hierzu gehören Pflanzen (z.B. rote Zwiebeln, Arnika oder Belladonna), Mineralien oder Tiere (z.B. tote Bienen). Als Darreichungsformen werden häufig sogenannte Globuli (kleine Zuckerkugeln) oder Lösungen verwendet, aber auch Salben, Gele oder Tabletten finden Anwendung.

[2]

Christian Friedrich Samuel Hahnemann (* 10. April 1755 in Meißen; † 2. Juli 1843 in Paris) war ein deutscher Arzt, medizinischer Schriftsteller und Übersetzer. Er ist der Begründer der Homöopathie.

**Naturheilkunde ≠ Homöopathie**

Oftmals werden Homöopathie und Naturheilkunde gleichgesetzt, doch dabei handelt es sich um grundsätzlich verschiedene Ansätze.

Die Naturheilkunde setzt natürliche Wirkstoffe und Reize ein. Zu ihr gehören Bewegungstherapie, Wasseranwendungen, Klimareize (z.B. ein Aufenthalt an der See), Ernährung oder die Behandlung mit pflanzlichen Wirkstoffen. [3] Ziel ist es, den Körper bei Krankheit durch natürliche Verfahren/Inhaltsstoffe zu unterstützen.

So wirkt beispielweise Spazieren im Wald nachweislich stressreduzierend und trägt zum allgemeinen Wohlbefinden bei. [4] Pflanzliche Wirkstoffe können zur Therapie von Krankheiten genutzt werden und sind wissenschaftlich gut belegt.

Im Unterschied dazu verwendet die klassische Homöopathie Verdünnungsstufen. Diese Verdünnungsstufen unterscheiden sich jedoch stark im Vergleich mit anderen Medikamenten.

**Beispiel:**

Johanniskraut (Einsatz bei leichten Depressionen)

1 Tablette:

**0,9g** Wirkstoff


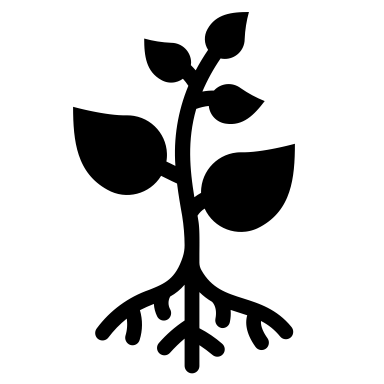


Handelsübliches Fläschchen Globuli

Ganzes Fläschchen:

**0,000 000 000 001 g** Wirkstoff

**Naturheilkunde**

**(pflanzliche Medikamente**)

**Homöopathie**

**(Globuli)**


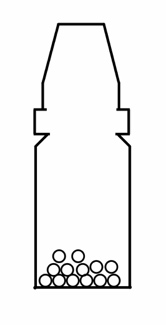


Arnica D12

**Eine Büroklammer = 1g**


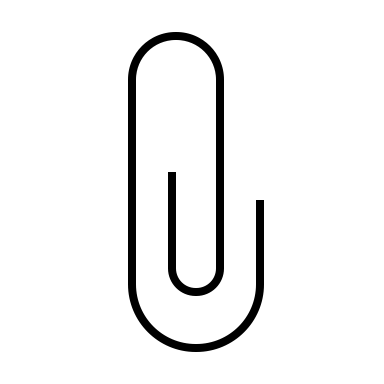


**Herstellung[5]**

Herstellung der Urtinktur

Im ersten Schritt werden zum Beispiel getrocknete Pflanzenteile für eine gewisse Zeit in Alkohol eingelegt. Dies führt dazu, dass Wirkstoffe extrahiert werden und die sogenannte Urtinktur entsteht (Ausgangslösung).

Verdünnung (Potenzierung)

Diese entstandene Urtinktur (Wirkstoff in Lösung) wird nun verdünnt. Die Verdünnungen werden in drei unterschiedlichen Kategorien durchgeführt. Die erstandenen Verdünnungsstufen werden als „Potenzen“ bezeichnet.

Wirksamkeit nimmt zu.

**D**- 1:10

**C-** 1:100

**Q/LM** 1:50.000

Je nachdem wie oft eine Verdünnung innerhalb einer Kategorie stattgefunden hat, wird diese Anzahl an den Buchstaben angehangen. So muss man zur Herstellung einer D1 Potenz 1 Teil Urtinktur und 9 Teile Ethanol/Wasser Gemisch verwenden. Für D10 würde man diesen Vorgang weitere neun Mal durchführen.

**Schüttelschlag:** Lösung wird zur Entwicklung der Kräfte gegen den Erdmittelpunkt geschüttelt.


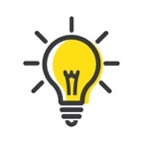


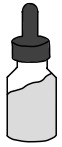


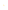


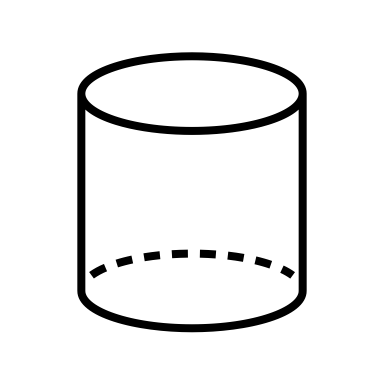

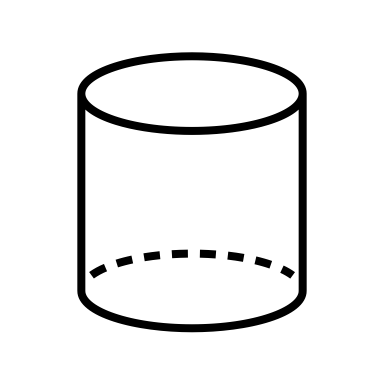

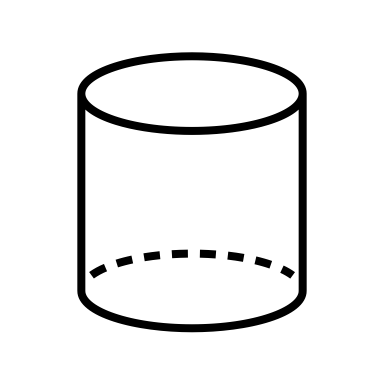

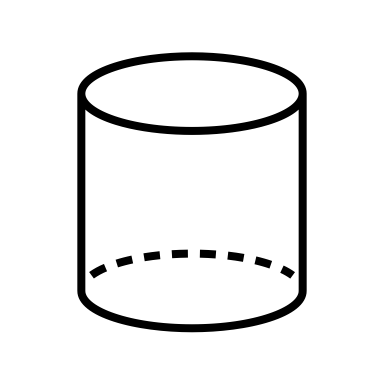


**Urtintkur**

**D1**

1 Teil Urtinktur

9 Teile Wasser/Alkohol

**D2**

1 Teil von D1

9 Teile Wasser/Alkohol

**D3**

1 Teil von D2

9 Teile Wasser Alkohol

**Verdünnung (Potenzierung)**

usw.

Zwischen diesen Verdünnungsstufen bzw. nach Fertigstellung dieser, werden jeweils 10 Schüttelschläge durchgeführt.


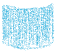

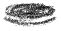

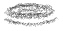

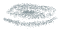

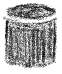

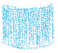

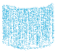


Herstellung Globuli

Diese oben beschriebene Verdünnung (Potenz) kann nun genutzt werden, um zum Beispiel Globuli herzustellen (kleine weiße Kugeln aus Zucker). Die am meisten verwendete Methode ist, die Kugeln mit der Verdünnung zu besprühen (Imprägnation), um somit eine gleichmäßige Verteilung zu erzielen.

**Zulassung und Registrierung homöopathischer Arzneimittel**

Wie es die Überschrift vermuten lässt, muss man bei diesem Thema zwei Begriffe voneinander unterscheiden: Registrierung und Zulassung.

Die meisten homöopathischen Arzneimittel werden nicht zugelassen, sondern registriert. [6]

In Zahlen ausgedrückt 🡪 **3508 registrierte und 1154 zugelassene Homöopathika** sind aktuell beim „Bundesinstitut für Arzneimittel und Medizinprodukte“ erfasst.

**Registrierung**

Um Homöopathische Arzneimittel zu registrieren und in den Handel zu bringen sind drei Kriterien zu erfüllen:

1. Der Hersteller gibt auf der Verpackung und in der Werbung kein Anwendungsgebiet an, behauptet also nicht, dass das Homöopathikum gegen bestimmte Krankheiten oder Beschwerden hilft.
2. Sie müssen über den Mund aufgenommen werden bzw. äußerlich angewendet werden
3. Mindestens um den Faktor 1:10.000 verdünnt sein

**Zulassung**

Möchte der Hersteller ein Anwendungsgebiet angeben (zum Beispiel „bei Fieber“), so muss das Homöopathikum als Arzneimittel zugelassen werden. Gängige Praxis ist es, die Wirksamkeit des Medikaments durch klinische Studien nachzuweisen. Dieser Prozess ist sehr aufwendig und kann bis zu zehn Jahre dauern. [7]

Homöopathika bilden hier eine Ausnahme. Solange es sich nicht um schwere oder lebensbedrohliche Krankheiten handelt, reicht es für die Zulassung oft aus, dass das Präparat bereits lange (mindestens seit 1978) so angewendet wird. Ein Wirksamkeitsnachweis bzw. eine Wirksamkeitsstudie wie oben beschrieben ist nicht erforderlich.

*„Bislang wurde noch kein homöopathisches Arzneimittel auf Basis einer solchen Studie zugelassen, so das Bundesinstitut für Arzneimittel und Medizinprodukte. Auf Nachfrage stellt sich allerdings heraus, dass auch noch kein Hersteller Ergebnisse von randomisierten klinischen Studien zur Zulassung vorgelegt hat.“[8]*

**Gibt es wissenschaftliche Untersuchungen zum Thema Homöopathie?**

*"Ein Wirkstoff, der nicht mehr da ist, kann nicht mehr wirken“ [9] (Zitat Helmholtz-Institut)*

Ja, die gibt es. Insgesamt gibt es drei systematische Übersichten [10-12], welche sich mit der Wirksamkeit von Homöopathie beschäftigen:

1997

2015

2017

Systematische Übersicht mit Meta-Analyse: Von 186 gefundenen Studien konnten am Ende 89 analysiert werden.

**Ergebnis:** Die Qualität der untersuchten Studien war oft unzureichend und es gibt keine eindeutigen Beweise, dass homöopathische Arzneimittel einem Placebo überlegen sind.

Systematische Übersicht: Ein Teil des australischen Gesundheitsministeriums (NHMRC) untersuchte insgesamt 215 Studien.

**Ergebnis:** Die Qualität der untersuchten Studien war oft unzureichend und es gibt keine eindeutigen Beweise, dass homöopathische Arzneimittel einem Placebo überlegen sind.

Systematische Übersicht: Analyse von 75 Studien zum Thema Wirksamkeit der Homöopathie mit dem Fokus auf hochwertige Studien.

**Ergebnis:** Die Qualität der untersuchten Studien war oft unzureichend und es gibt keine eindeutigen Beweise, dass homöopathische Arzneimittel einem Placebo überlegen sind.

**Fazit: Zusammenfassend lässt sich festhalten, dass die Wirksamkeit der Homöopathie nicht nachgewiesen werden konnte. Kein Präparat schafft, es einem Placebo überlegen zu sein.**

**Begriffe[13]:**

Im Rahmen einer systematischen Übersicht können die Ergebnisse aller gefundenen Studien unter bestimmten Umständen zu einem Gesamtergebnis zusammengefasst werden. Dies nennt man eine
**Meta-Analyse.** Das Gesamtergebnis hat oft eine deutlich höhere Aussagekraft als die Ergebnisse der Einzelstudien.

Eine **systematische Übersicht (engl. = systematic review)** fasst die Ergebnisse aller Studien zu einer medizinischen Behandlung zusammen, prüft ihre Qualität und analysiert sie nach einem festgelegten, methodisch durchdachten Prozess. Sie ist eine „Studie über Studien“. Wenn sie gut gemacht ist, kann sie einen verlässlichen Überblick über den Stand des Wissens zu einem Thema geben.

**Placebo und Homöopathie**

Der Begriff Placebo taucht häufig im Zusammenhang mit Homöopathie auf, da die beobachteten Wirkungen häufig auf den Placeboeffekt zurückzuführen sind. Was ist also ein Placebo und wie kommt der Effekt zu Stande? [14]

Ein Placebo (von lateinisch placebo „ich werde gefallen“) oder Scheinmedikament ist ein Arzneimittel, das meist keinen Arzneistoff enthält und somit auch keine Arzneimittel- Wirkung hat.

Durch die Verabreichung eines solchen „Scheinmedikaments“ kommt es zum sogenannten Placebo-Effekt. Dabei spielen zwei Mechanismen eine wichtige Rolle:

**Erfahrung**: „Die Tablette hat schon mal geholfen“

**Erwartung**: „Sie wird bestimmt auch jetzt helfen“


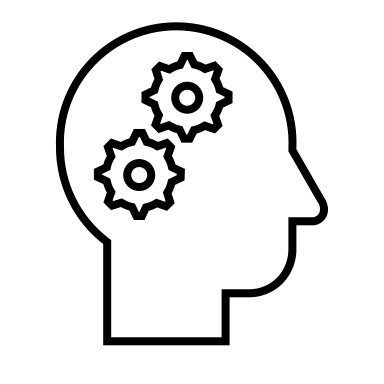

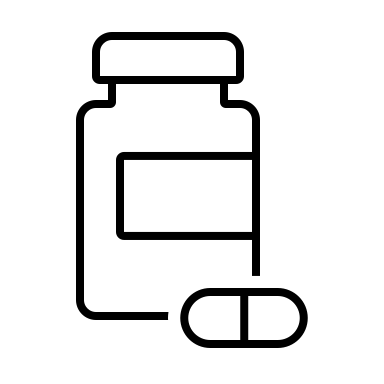


So kann bereits die Erwartung, ein Schmerzmedikament einzunehmen, dazu beitragen, dass der schmerzlindernde Effekt verstärkt wird. [15]

*Wie kann das aber sein, wenn doch kein Wirkstoff enthalten ist?*

Allein positive Erwartungen können im Körper dafür sorgen, dass unser emotionales Denken verändert wird. Der Gedanke an ein Treffen mit Freunden erzeugt beispielweise ein besseres Gefühl als ein Treffen mit dem Chef. Gebiete im Gehirn werden aktiviert, die sich positiv auf unser Krankheitsbefinden auswirken. [16]

Deshalb kann es unter homöopathischen Behandlungen zu scheinbaren Verbesserungen kommen. Patienten nehmen eine Verminderung von Symptomen wahr. Auch Selbstheilungskräfte und die natürlichen Entwicklungen einer Krankheit können dazu führen, dass eine Krankheit oder Beschwerden besser werden. So endet eine Erkältungskrankheit häufig nach wenigen Tagen. Patienten/-innen, die in der Zeit homöopathische Mittel eingenommen haben, glauben oft, dass die Besserung an diesen Mitteln liegen würde.

**Homöopathie bei Krebs?**

Viele Patienten/-innen möchten während einer Krebstherapie zusätzlich aktiv werden, um die Therapie zu unterstützen, sich zu stärken und Beschwerden zu lindern. [17]

Dabei überlegen einige, ob ihnen Homöopathie helfen kann. Deswegen soll im Folgenden kurz erklärt werden, welche Wirkungen für die Homöopathie in der Behandlung von Krebserkrankungen untersucht wurden.

In der **deutschen Leitlinie [18, 19]** wurden sechs Studien analysiert, welche sich mit dem Thema Homöopathie bei Krebs beschäftigen.

**Leitlinien in der Medizin**

„Sie werden als Handlungsempfehlungen nach einer bestimmten Methodik entwickelt und geben den Erkenntnisstand der Medizin zu einem bestimmten Zeitpunkt wieder.“

🡪 Experten der jeweiligen Fachgebiete verfassen diese Leitlinien, was eine hohe wissenschaftliche Qualität sichert.

Keine der Studien konnte eine Wirksamkeit der homöopathischen Arzneimittel in der Tumortherapie nachweisen. Alle untersuchten Studien weisen Mängel auf und eignen sich nicht für eine eindeutige Empfehlung in der Krebstherapie.

**Solange es nicht schadet?**

Homöopathische Arzneimittel werden oft als „schonend“ und frei von Nebenwirkungen dargestellt“. Bei niedrigen Potenzen sind Nebenwirkungen durchaus möglich [20], da noch Wirkstoff enthalten ist.

Gefährlicher sind jedoch indirekte Folgen. Wenn Patienten auf Homöopathie vertrauen, statt eine wirksame Therapie zu machen, kann es bei schwerwiegenden Erkrankungen wie Krebs zu schweren Schäden kommen, wenn der Beginn einer wirksamen Therapie verzögert oder eine wirksame Therapie unterbrochen wird. [21, 22]

Es ist auch ein Schaden für Patienten/-innen, wenn sie auf wirksame Medikamente gegen Nebenwirkungen der Krebstherapie verzichten, weil sie hoffen, dass Homöopathie ihnen helfen kann.

**Werden die Kosten für Homöopathie von der Kasse übernommen?**

Grundsätzlich gehört Homöopathie nicht zum Leistungskatalog der gesetzlichen Krankenkassen. Trotzdem werden die Kosten von manchen Versicherungen getragen.

Ähnlich wie bei der Zulassung benötigt es auch hier keinen wissenschaftlichen Nachweis der Wirkung.

Die Kosten für die Homöopathie bei den gesetzlichen Krankenkassen belaufen sich auf ca. 20 Millionen Euro im Jahr (Stand 2019). [23]

Etwa 2/3 der Kassen sind daran beteiligt. Die Aussage, dass durch homöopathische Behandlungen Kosten eingespart werden können, [24-26], wurde 2015 widerlegt. [27] Im Vergleich zur üblichen Versorgung, konnte gezeigt werden, dass eine zusätzliche homöopathische Behandlung mit deutlich höheren Kosten verbunden ist. In anderen europäischen Ländern wie Frankreich oder England werden dies Kosten von den Krankenkassen nicht erstattet. (In Schweden war homöopathische Arznei bis 2011 sogar gänzlich verboten.)

**Literatur**

1. NIH. *Homeopathy: What You Need to Know*. 2021 [cited 2021 11.11]; Available from: <https://www.nccih.nih.gov/health/homeopathy>.

2. Wikipedia. *Samuel Hahnemann*. 2022; Available from: <https://de.wikipedia.org/wiki/Samuel_Hahnemann>.

3. DocCheckFlexikon. *Naturheilkunde*. 2021 [cited 2021 06.09]; Available from: <https://flexikon.doccheck.com/de/Naturheilkunde>

4. Hunter, M.R., B.W. Gillespie, and S.Y. Chen, *Urban Nature Experiences Reduce Stress in the Context of Daily Life Based on Salivary Biomarkers.* Front Psychol, 2019. **10**: p. 722.

5. DeutscherApothekerVerlag, *Homöopathisches Arzneibuch* Vol. 13. 2021.

6. BfArM. *Statistik „Besondere Therapieeinrichtungen und Tradionelle Arzneimittel“*. 2021 [cited 2021 01.11]; Available from: <https://www.bfarm.de/DE/Aktuelles/Statistiken/AM_statistik/Besondere_Therapierichtungen_statistik/statistik-bescheidzahlen.html;jsessionid=F1DCAAF35A35AB1E68E2A1CF583353CD.intranet672?nn=936914>.

7. ScienceMediaCenterGermany. *Arzneimittel: Von der Entwicklung bis zur Zulassung*. 2017 [cited 2021 11.09]; Available from: <https://www.sciencemediacenter.de/alle-angebote/fact-sheet/details/news/arzneimittel-von-der-entwicklung-bis-zur-zulassung/>.

8. Quarks. *So fragwürdig ist die Zulassung von homöopathsichen Arzneimitteln*. 2019 [cited 2021 10.11]; Available from: <https://www.quarks.de/gesundheit/medizin/darum-ist-bei-vielen-homoeopathischen-mitteln-keine-wirkung-nachgewiesen/>.

9. HelmholtzInstitut. *Wirkt Homöopathie wirklich?* 2019; Available from: <https://www.helmholtz.de/gesundheit/wirkt-homoeopathie-wirklich/>.

10. Linde, K., et al., *Are the clinical effects of homeopathy placebo effects? A meta-analysis of placebo-controlled trials.* Lancet, 1997. **350**(9081): p. 834-43.

11. Mathie, R.T., et al., *Randomised, double-blind, placebo-controlled trials of non-individualised homeopathic treatment: systematic review and meta-analysis.* Syst Rev, 2017. **6**(1): p. 63.

12. NHMRC. *Homeopathy*. 2021 [cited 2021 14.09]; Available from: <https://www.nhmrc.gov.au/about-us/resources/homeopathy>.

13. gesundheitsinformation.de. *Was sind systematische Übersichten und Meta-Analysen?* 2020 [cited 2021 12.11]; Available from: <https://www.gesundheitsinformation.de/was-sind-systematische-uebersichten-und-meta-analysen.html>.

14. Wikipedia. *Placebo*. 2021 [cited 2021 15.09]; Available from: <https://de.wikipedia.org/wiki/Placebo>.

15. Bingel, U., et al., *The effect of treatment expectation on drug efficacy: imaging the analgesic benefit of the opioid remifentanil.* Sci Transl Med, 2011. **3**(70): p. 70ra14.

16. Benedetti, F., E. Carlino, and A. Pollo, *How placebos change the patient's brain.* Neuropsychopharmacology, 2011. **36**(1): p. 339-54.

17. Huebner, J., et al., *User rate of complementary and alternative medicine (CAM) of patients visiting a counseling facility for CAM of a German comprehensive cancer center.* Anticancer Res, 2014. **34**(2): p. 943-8.

18. bundesaerztekammer.de. *Verbindlichkeit von Richtlinien, Leitlinien, Empfehlungen und Stellungnahmen*. [cited 2021 03.10]; Available from: <https://www.bundesaerztekammer.de/richtlinien/>.

19. AWMF. *S3-Leitlinie Komplementärmedizin in der Behandlung von onkologischen PatientInnen*. 2021 [cited 2021 12.11]; Available from: <https://www.leitlinienprogramm-onkologie.de/fileadmin/user_upload/Downloads/Leitlinien/Komplement%C3%A4r/Version_1/LL_Komplement%C3%A4r_Langversion_1.0.pdf>.

20. Posadzki, P., A. Alotaibi, and E. Ernst, *Adverse effects of homeopathy: a systematic review of published case reports and case series.* Int J Clin Pract, 2012. **66**(12): p. 1178-88.

21. Johnson, S.B., et al., *Use of Alternative Medicine for Cancer and Its Impact on Survival.* J Natl Cancer Inst, 2018. **110**(1).

22. aerzteblatt.de. *Alternative Medizin: Keine Alternative bei Krebs*. 2020 [cited 2021 15.09]; Available from: <https://www.aerzteblatt.de/archiv/212881/Alternative-Medizin-Keine-Alternative-bei-Krebs>.

23. aerzteblatt.de. *Spahn will Homöopathie auf Kassenkosten nicht antasten*. 2019 [cited 2021 10.11]; Available from: <https://www.aerzteblatt.de/nachrichten/106083/Spahn-will-Homoeopathie-auf-Kassenkosten-nicht-antasten>.

24. Baars, E.W. and P. Kooreman, *A 6-year comparative economic evaluation of healthcare costs and mortality rates of Dutch patients from conventional and CAM GPs.* BMJ Open, 2014. **4**(8): p. e005332.

25. Ernst, E., *Article by A Jain: Does homeopathy reduce the cost of conventional drug prescribing?* Homeopathy, 2003. **92**(4): p. 233; author reply 233.

26. Studer, H.P. and A. Busato, *Comparison of Swiss basic health insurance costs of complementary and conventional medicine.* Forsch Komplementmed, 2011. **18**(6): p. 315-20.

27. Ostermann, J.K., T. Reinhold, and C.M. Witt, *Can Additional Homeopathic Treatment Save Costs? A Retrospective Cost-Analysis Based on 44500 Insured Persons.* PLoS One, 2015. **10**(7): p. e0134657.

**Impressum**

Prof. Dr. med. Jutta Hübner

Maximilian Gimbel

# Stiftung Perspektiven

Adenauerallee 87, 53113 Bonn

Telefon: (0228) 33 88 9 -215

Fax: (0228) 33 88 9 -222

E-Mail: [info@stiftung-perspektiven.de](mailto:info@stiftung-perspektiven.de)
