# Supplementary material for: Efficacy of a Decision‐Making Aid About Homeopathy in Patients With Cancer: A Single‐Arm, Pre–Post Observational Study
Source: Health Sci Rep. 2026 Jul 30;9(8):e72943. doi: 10.1002/hsr2.72943 (PMC13420187; doi:10.1002/hsr2.72943)
Supplement: Supplementary file 2 — Supporting File 2 [file HSR2-9-e72943-s002.docx]

**Fragebogen zur Entscheidungshilfe Homöopathie**

Liebe/r Teilnehmer/in,

mein Name ist Maximilian Gimbel, ich studiere Medizin an der FSU Jena und beschäftige mich im Rahmen meiner Doktorarbeit mit dem Thema Homöopathie. Gemeinsam mit Fr. Prof. Dr. med. Hübner habe ich eine Entscheidungshilfe zu diesem Thema entworfen, welche nun mit Ihrer Hilfe getestet werden soll. Der Fragebogen umfasst 28 Fragen und die Entscheidungshilfe 13 Seiten. Der erste Teil der Fragen soll vor dem Lesen der Entscheidungshilfe beantwortet werden, der Zweite danach. Insgesamt wird die Bearbeitung für Sie 30-45 Minuten in Anspruch nehmen. Für den Erfolg dieser Studie ist es wichtig, dass Sie den Fragebogen vollständig ausfüllen. Alle Daten werden anonym erhoben und streng vertraulich behandelt.

1. Ich erkläre mich einverstanden, dass im Rahmen dieser Befragung anonyme Daten über mich erhoben und in Papierform aufbewahrt, sowie auf elektronischen Datenträgern passwortgeschützter Rechner zur wissenschaftlichen Auswertung gespeichert werden.

2. Außerdem erkläre ich mich einverstanden, dass autorisierte und zur Verschwiegenheit verpflichtete zuständige Überwachungsbehörden in die anonymisierten Daten Einsicht nehmen, soweit dies für die Überprüfung der ordnungsgemäßen Durchführung der Studie notwendig ist.

3. Ich bin darüber aufgeklärt, dass ich an der Befragung freiwillig teilnehme und mein Einverständnis erteile. Die Einwilligung zur Erhebung und Verarbeitung meiner anonymen Daten ist unwiderruflich. Aufgrund der anonymen Erhebung der Daten können die gespeicherten Daten rückwirkend nicht mehr gelöscht werden.

4. Ich erkläre mich damit einverstanden, dass meine Daten nach Beendigung der Befragung mindestens zehn Jahre aufbewahrt werden. Danach werden meine Daten gelöscht, soweit nicht gesetzliche oder satzungsmäßige Aufbewahrungsfristen entgegenstehen.

Durch Ausfüllen und Rückgabe des Fragebogens stimmen Sie der Teilnahme an der Befragung zu.

Vielen Dank für Ihre Teilnahme!

**Auszufüllen vor dem Lesen der Entscheidungshilfe**

1) Geschlecht: ⭘ männlich ⭘ weiblich ⭘ divers

2) Alter: ______ (Bitte eintragen)

3) Familienstand:

⭘ ledig

⭘ verheiratet/ in Lebenspartnerschaft lebend

⭘ verwitwet/ Lebenspartner verstorben

⭘ Geschieden/Lebenspartnerschaft aufgehoben

4) Höchster Schulabschluss (Bitte kreuzen Sie an.):

⭘ noch in schulischer Ausbildung

⭘ Haupt-(Volks-)schulabschluss

⭘ Abschluss der polytechnischen Oberschule

⭘ Mittlerer Abschluss

⭘ Fachhochschul- oder Hochschulreife

5) Höchster Bildungsabschluss (Bitte kreuzen Sie an.):

⭘ abgeschlossene Berufsausbildung

⭘ abgeschlossenes Studium

6) Unten sehen Sie eine Skala von 1-10, die zeigen soll, wo die Menschen in Deutschland stehen. Ganz oben stehen die Menschen mit dem meisten Geld, der höchsten Bildung und den besten Jobs (10). Ganz unten diejenigen mit dem wenigsten Geld, der niedrigsten Bildung und den schlechtesten Jobs bzw. ohne Jobs (1). Wo würden Sie sich platzieren? (Bitte kreuzen Sie an.)

⭘ 1 ⭘ 2 ⭘ 3 ⭘ 4 ⭘ 5 ⭘ 6 ⭘ 7 ⭘ 8 ⭘ 9 ⭘ 10

7) Können Sie sich unter dem Begriff Homöopathie etwas vorstellen? (Bitte kreuzen Sie an.)

⭘ Ja, ich kenne die Grundlagen der Homöopathie genau

⭘ Ich kenne die Grundlagen der Homöopathie ungefähr

⭘ Ich habe den Begriff schon mal gehört

⭘ Nein, ich kann mir nichts darunter vorstellen

8) In der Homöopathie wird das Verfahren der Potenzierung eingesetzt. (Bitte kreuzen Sie an.)

Mit steigender Verdünnung wird die Wirksamkeit:

⭘ höher

⭘ geringer

⭘ gleichbleibend

9) Haben Sie in der Vergangenheit Homöopathie genutzt? (Bitte kreuzen Sie an.)

⭘ Ja

⭘ Nein

Wenn ja:

⭘ Unabhängig von einer Krebserkrankung

⭘ Während einer Krebserkrankung

Gegen welche Beschwerden haben sie Homöopathie genutzt?

___________________________________________________

___________________________________________________

10) Wie viel Geld wären Sie bereit für homöopathische Arzneimittel (pro Monat) auszugeben?

(Bitte kreuzen Sie an.)

⭘ 0 – 20€

⭘ 20 – 40€

⭘ 40 – 60€

⭘ 60 – 80€

⭘ über 80€

11) Im Folgenden sind Krankheitsbilder aufgelistet. Bitte bewerten Sie mit Hilfe der Skala, inwiefern Homöopathie bei diesen eingesetzt werden kann. (Zutreffendes ankreuzen)

|  | Sollte eingesetzt werden. | Kann eingesetzt werden. | Sollte eher nicht eingesetzt werden. | Sollte auf keinen Fall eingesetzt werden. | Ich weiß nicht. |
| --- | --- | --- | --- | --- | --- |
| **Seelische und psychische Belastungen** |  |  |  |  |  |
| **Grippaler Infekt mit Fieber** |  |  |  |  |  |
| **Schlaganfall** |  |  |  |  |  |
| **bei akuten Schmerzen in der Brust** |  |  |  |  |  |
| **Gelenkbeschwerden** |  |  |  |  |  |
| **Krebserkrankung** |  |  |  |  |  |

12) Wie würden Sie Homöopathie zur Krebsbehandlung einsetzen? (Bitte kreuzen Sie an.)

⭘ Zusätzlich zur klassischen Krebstherapie (Chemotherapie, Bestrahlung etc.)

⭘ Als alleinige Therapie

⭘ Ich weiß es nicht

13) Denken Sie, dass ein homöopathisches Mittel Nebenwirkungen haben kann? (Bitte kreuzen Sie an.)

⭘ Ja

⭘ Nein

⭘ Ich weiß es nicht

14) Können Sie sich unter dem Begriff Placebo etwas vorstellen? (Bitte kreuzen Sie an.)

⭘ Ja, ich kenne mich damit aus

⭘ Ja, ich kann mir unter dem Begriff ungefähr etwas vorstellen

⭘ Ich habe den Begriff schon mal gehört

⭘ Nein, ich kann mir nichts darunter vorstellen

**Auszufüllen nach dem Lesen der Broschüre**

15) Ich fühle mich durch die Entscheidungshilfe gut über Homöopathie aufgeklärt. (Bitte kreuzen Sie an.)

⭘ trifft voll zu ⭘ trifft eher zu ⭘ trifft teilweise zu ⭘ trifft eher nicht zu ⭘ trifft gar nicht zu

16) Ich konnte mein Wissen über Homöopathie durch die Entscheidungshilfe erweitern. (Bitte kreuzen Sie an.)

⭘ trifft voll zu ⭘ trifft eher zu ⭘ trifft teilweise zu ⭘ trifft eher nicht zu ⭘ trifft gar nicht zu

17) Die Entscheidungshilfe hat meine Meinung zur Homöopathie verändert. (Bitte kreuzen Sie an.)

⭘ trifft voll zu ⭘ trifft eher zu ⭘ trifft teilweise zu ⭘ trifft eher nicht zu ⭘ trifft gar nicht zu

18) Welche (neuen) Informationen waren für Sie von besonderem Interesse?

⭘ Herstellungsverfahren (Potenzierung)

⭘ Zulassungsverfahren

⭘ Wissenschaftliche Untersuchungen

⭘ Anderes: ____________________

19) Eine homöopathische Therapie kommt nach Lesen der Entscheidungshilfe für mich in Frage.

(Bitte kreuzen Sie an.)

⭘ trifft voll zu ⭘ trifft eher zu ⭘ trifft teilweise zu ⭘ trifft eher nicht zu ⭘ trifft gar nicht zu

20) Denken Sie, dass ein homöopathisches Mittel Nebenwirkungen haben kann? (Bitte kreuzen Sie an.)

⭘ Ja

⭘ Nein

⭘ Ich weiß es nicht

21) Können Sie sich unter dem Begriff Placebo etwas vorstellen? (Bitte kreuzen Sie an.)

⭘ Ja, ich kenne mich damit aus

⭘ Ja, ich kann mir unter dem Begriff ungefähr etwas vorstellen

⭘ Ich habe den Begriff schon mal gehört

⭘ Nein, ich kann mir nichts darunter vorstellen

22) In der Homöopathie wird das Verfahren der Potenzierung eingesetzt. (Bitte kreuzen Sie an.)

Mit steigender Verdünnung wird die Wirksamkeit:

⭘ höher

⭘ geringer

⭘ gleichbleibend

23) Im Folgenden sind Krankheitsbilder aufgelistet. Bitte bewerten Sie mit Hilfe der Skala, inwiefern Homöopathie bei diesen eingesetzt werden kann. (Zutreffendes ankreuzen)

|  | Sollte eingesetzt werden. | Kann eingesetzt werden. | Sollte eher nicht eingesetzt werden. | Sollte auf keinen Fall eingesetzt werden. | Ich weiß nicht. |
| --- | --- | --- | --- | --- | --- |
| **Seelische und psychische Belastungen** |  |  |  |  |  |
| **Grippaler Infekt mit Fieber** |  |  |  |  |  |
| **Schlaganfall** |  |  |  |  |  |
| **bei akuten Schmerzen in der Brust** |  |  |  |  |  |
| **Gelenkbeschwerden** |  |  |  |  |  |
| **Krebserkrankung** |  |  |  |  |  |

24) Die Broschüre war für mich verständlich formuliert. (Bitte kreuzen Sie an.)

⭘ trifft voll zu ⭘ trifft eher zu ⭘ trifft teilweise zu ⭘ trifft eher nicht zu ⭘ trifft gar nicht zu

25) Die Abbildungen in der Broschüre haben mir beim Verständnis geholfen. (Bitte kreuzen Sie an.)

⭘ trifft voll zu ⭘ trifft eher zu ⭘ trifft teilweise zu ⭘ trifft eher nicht zu ⭘ trifft gar nicht zu

26) Der Umfang der Informationen war: (Bitte kreuzen Sie an.)

⭘ zu umfangreich ⭘ genau richtig ⭘ zu gering

27) Würden Sie die Entscheidungshilfe weitergeben? (Bitte kreuzen Sie an.)

⭘ Ja

⭘ Nein

28) Die Entscheidungshilfe stellt die wissenschaftlichen Erkenntnisse neutral dar und gibt einen Überblick über die Homöopathie.

⭘ trifft voll zu ⭘ trifft eher zu ⭘ trifft teilweise zu ⭘ trifft eher nicht zu ⭘ trifft gar nicht zu

Das möchte ich noch anmerken:

___________________________________________________________________________________________

___________________________________________________________________________________________

___________________________________________________________________________________________
